# Comparing children and adults with medulloblastoma: a SEER based analysis

**DOI:** 10.18632/oncotarget.23773

**Published:** 2018-07-10

**Authors:** Qian Li, Zhenguo Dai, Yuze Cao, Lihua Wang

**Affiliations:** ^1^ Department of Neurology, The Second Affiliated Hospital, Harbin Medical University, Harbin 150081, Heilongjiang, China; ^2^ Department of Cardiology, The Second Affiliated Hospital, Harbin Medical University, Harbin 150081, Heilongjiang, China

**Keywords:** medulloblastoma, SEER, survival, treatment, prognostic factors

## Abstract

Medulloblastoma (MB) is a brain malignancy, which commonly occurs in children, but is rare in adults. The Surveillance, Epidemiology, and End Results (SEER) database was used to compare survival, clinical features, and prognostic factors of children and adults with MB from 1992 to 2013. Overall survival estimates were compared using the Kaplan–Meier method, and Cox Proportion Hazard Regression modeling was used to evaluate prognostic variables. We identified 616 children (63.8%) and 349 adults (36.2%) with diagnosis of MB. The estimated survival rates for children diagnosed with MB for 2, 5, and 10 years were 85.6%, 75.5%, and 67.9%, respectively; the corresponding estimates for adults were 84.9%, 74.2%, and 67.3%. Radiotherapy was the only identical prognostic factor observed in the two groups. Children MB patients were more likely to experience distal metastases that was associated with increased hazard of mortality, and be diagnosed after 2003. Among adult MB patients, gross total resection (GTR) was a favorable prognostic factor, while large cell/anaplastic (LC/A) histology was correlated with decreased survival. Our analysis highlighted that both groups had similar overall survival time, but the prognostic factors were not comparable, except radiotherapy which was associated with better survival.

## INTRODUCTION

Medulloblastoma (MB) is a highly malignant neuroectodermal tumors, that belongs to high-grade glioma (HGG) and was categorized into WHO grade IV tumours of the Central Nervous System (CNS) [1]. MB commonly occurs among children, accounting for approximately 30% of pediatric CNS neoplasms; however, it is a rare disease among adults, with an annual incidence rate of 0.05 per 100,000 per year, which is fewer than 3% of all the primary neoplasms of the CNS [2, 3].

Controversy remains regarding the survival of MB between children and adults patients, some data suggests that adults fare better than children [4]. Emily K *et al.* reported that children and adults with MB do not differ with respect to overall survival, yet patients who are 3 years old or less fare significantly worse [5]. However, Rose Lai *et al.* suggested that the survival of children, especially older than 3 years of age, may be better than adults [6]. Furthermore, there has been no research that directly compare and evaluate the overall survival between children (4–19 years) with those of adults (≥ 20 years).

In children, the sample size was adequate in determining the clinical prognostic factors that guide the therapeutic strategies [7]; whereas in adults, the disease rarely occurred. The majority of reported survival rates and prognostic factors were based on single-institution comprising small series [8–11], or clinical studies that group together adult MB children MB, thus, the results may be inconclusive or may not permit a definite assessment of the prognostic role of clinical and pathologic factors to guide the therapy for adults. Previous studies suggested it is reasonable to consider grouping adults with children in clinical trials for MB [5]. However, there have been debates as to whether adults have a similar therapeutic response with that of children, such as radiotherapy or extent of surgery.

The Surveillance, Epidemiology and End Results (SEER) program of the National Cancer Institute (NCI) could provide us with sufficient amount of patients who are representative of the US population, without selection biases. In this study, we aimed to determine whether adults and children with MB differ in survival, and to evaluate the prognostic factors or treatment efficacy in pediatric versus adult patients on a national level by using SEER database.

## RESULTS

### Patient characteristics

**Figure 1:**
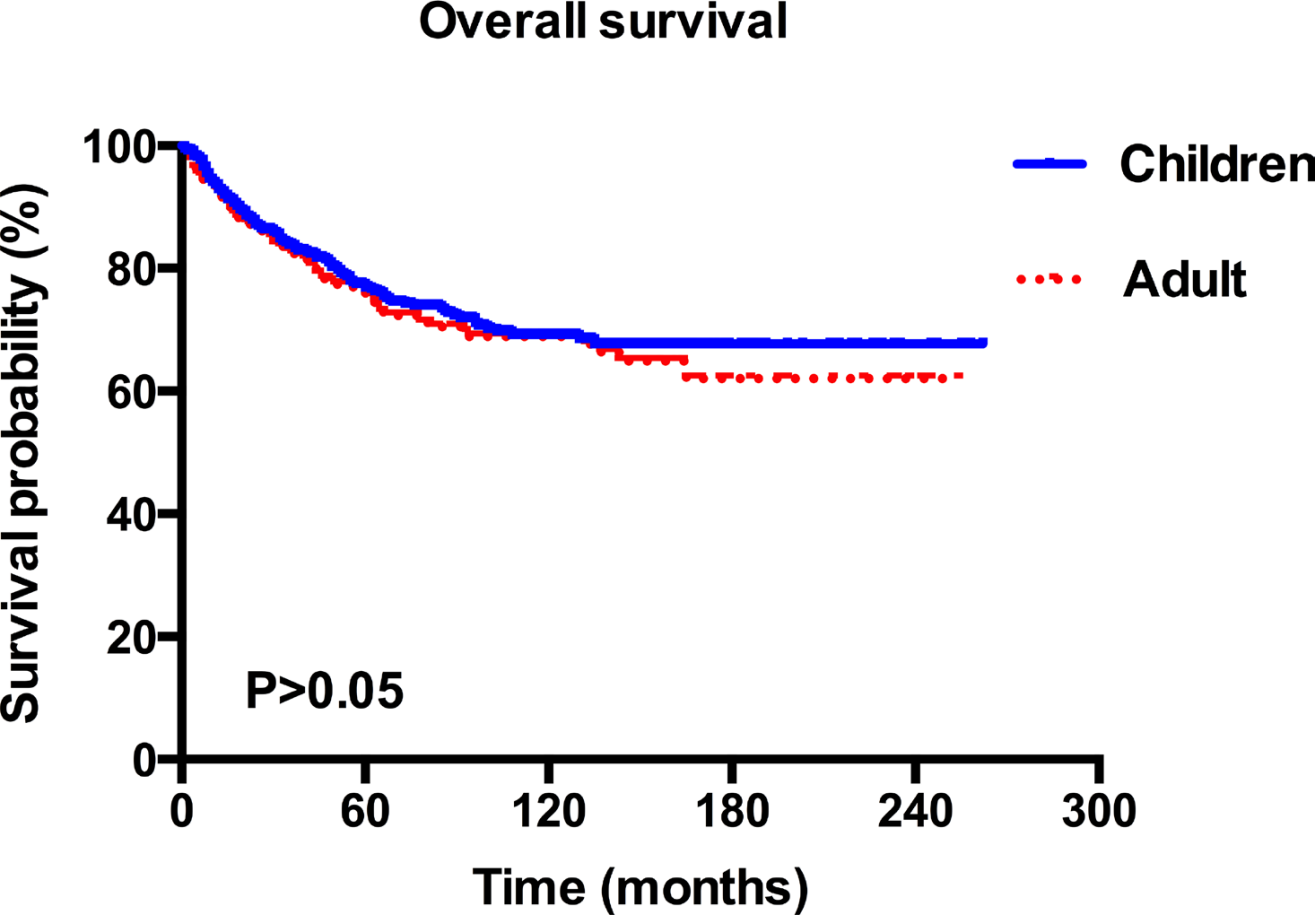
Kaplan–Meier overall survival curves for children and adult patients with MB

**Figure 2:**
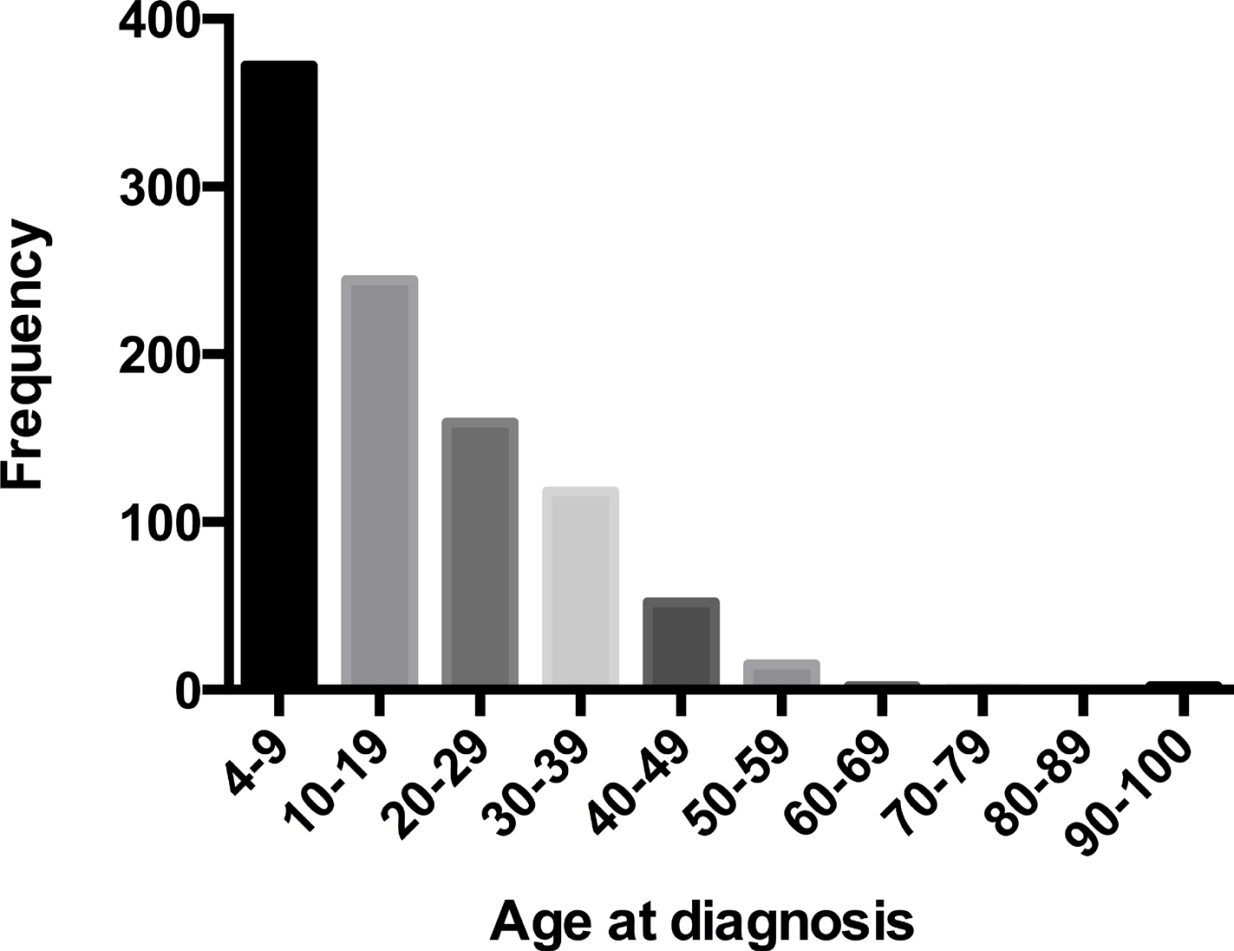
Patients distribution by age at diagnosis in 965 patients with MB, diagnosed from 1992 to 2013

A total of 965 MB worth of data on patients was analyzed in this study. Among these patients, 616 (63.8%) were children and 349 (36.2%) were adults. The median age was 8 years old for children and 31 years old for adults. The estimated 2- 5- and 10-year overall survival for children were 85.6%, 75.5%, and 67.9%, respectively, and the corresponding estimates for adults were 84.9%, 74.2%, and 67.3% (Figure 1). Epidemiological data showed that in the paediatric age group, MB occurred commonly during the first decade (38.5%) and in adults, the third decade (16.5%). The peak age of onset for MB patients was in those 9 years old and younger, after which the frequency appears to show an exponential decrease with age (Figure 2).

The majority of patients are white (85.4%), and 61.5% of which are male. About 9.6% of histopathology was the desmoplastic nodular type, and 4.8% had the large cell /anaplastic variant. The gross total resection was determined for 59.0% patients, and over 80% of patients had radiotherapy. The majority of tumor (92.1%) located in infratentorial. About 11.7% of patients had spinal cord, cerebrospinal fluid (CSF), or extra neural metastases at the time of diagnosis. While comparing with adult MB patients, children MB patients were more likely to exhibit distal metastases (CSF, spinal cord or extra neural) (14.9% vs 6.0%), and slightly more often to be diagnosed in 2003–2013 (75.5% vs 69.3%). However, the characteristics and distribution of the two groups showed no significant difference concerning sex, race, histological type, tumor size, extent of surgery, radiotherapy, and site. A detailed listing of the patients’ clinical characteristics was presented in Table 1.

**Table 1:** Demographic, tumor, and treatment characteristics

	All patients		Children MB		Adult MB		*P* value
	*N*	%	*N*	%	*N*	%	
**No. of patients**	965	100	616	63.8	349	36.2	<0.001
**Age**							
**Median**			8		31		<0.001
**Interquartile range (years)**	4–100		4–19		20–100		
**Sex**							
**Male**	593	61.5	381	61.9	212	60.7	0.735
**Female**	372	38.5	235	38.1	137	39.3	
**Race**							
**White**	824	85.4	518	84.1	306	87.7	0.315
**Non-white**	138	14.3	96	15.6	42	12.0	
**Unknown**	3	0.3	2	0.3	1	0.3	
**Marital status**							
**Married**	159	16.5	2	0.3	157	45.0	<0.001
**Unmarried**	794	82.3	613	99.5	181	51.9	
**Unknown**	12	1.2	1	0.2	11	3.2	
**Histology**							
**Medulloblastoma**	822	85.2	524	85.1	298	85.4	0.208
**Desmoplastic nodular medulloblastoma**	93	9.6	54	8.8	39	11.2	
**Medullomyoblastoma**	4	0.4	3	0.5	1	0.3	
**Large cell /Anaplastic medulloblastoma**	46	4.8	35	5.7	11	3.2	
**Tumor size (mm)**							
**≤40**	392	40.6	234	38	158	45.3	0.086
**>40**	371	38.4	247	40.1	124	35.5	
**Unknown**	202	20.9	135	21.9	67	19.2	
**Treatment protocol**							
**No treatment**	12	1.2	9	1.5	3	0.9	0.078
**Surgery only**	121	12.5	72	11.7	49	14.0	
**Radiation only**	7	0.7	4	0.6	3	0.9	
**Surgery and radiation**	802	83.1	522	84.7	280	80.2	
**Unknown**	23	2.4	9	1.5	14	4	
**Extent of resection**							
**NS**	21	2.2	14	2.3	7	2.0	0.127
**GTR**	569	59.0	377	61.2	192	55	
**STR**	367	38.0	222	36.0	145	41.5	
**Unknown**	8	0.8	3	0.5	5	1.4	
**Radiation**							
**RT**	817	84.7	528	85.7	289	82.8	0.268
**No RT**	133	13.8	81	13.1	52	14.9	
**Unknown**	15	1.6	7	1.1	8	2.3	
**Year of diagnosis**							
**1992–2002**	258	26.7	151	24.5	107	30.7	0.038
**2003–2013**	707	73.3	465	75.5	242	69.3	
**Site**							
**Supratentorial**	44	4.6	31	5	13	3.7	0.140
**Infratentorial**	889	92.1	560	90.9	329	94.3	
**Others**	32	3.3	25	4.1	7	2	
**Extent of disease at diagnoses**							
**Diseases confined to the brain**	833	86.3	510	82.8	323	92.6	<0.001
**Distal metastases (CSF, spinal cord or extraneural)**	113	11.7	92	14.9	21	6.0	
**Unknown**	19	2	14	2.3	5	1.4	
**Vital status**							
**Living**	725	75.1	468	76.0	257	73.6	0.420
**Dead**	240	24.9	148	24.0	92	26.4	

Abbreviations: GTR: gross total resection; STR: subtotal resection; NS: no surgery; RT: radiotherapy.

### Univariate analysis

**Figure 3:**
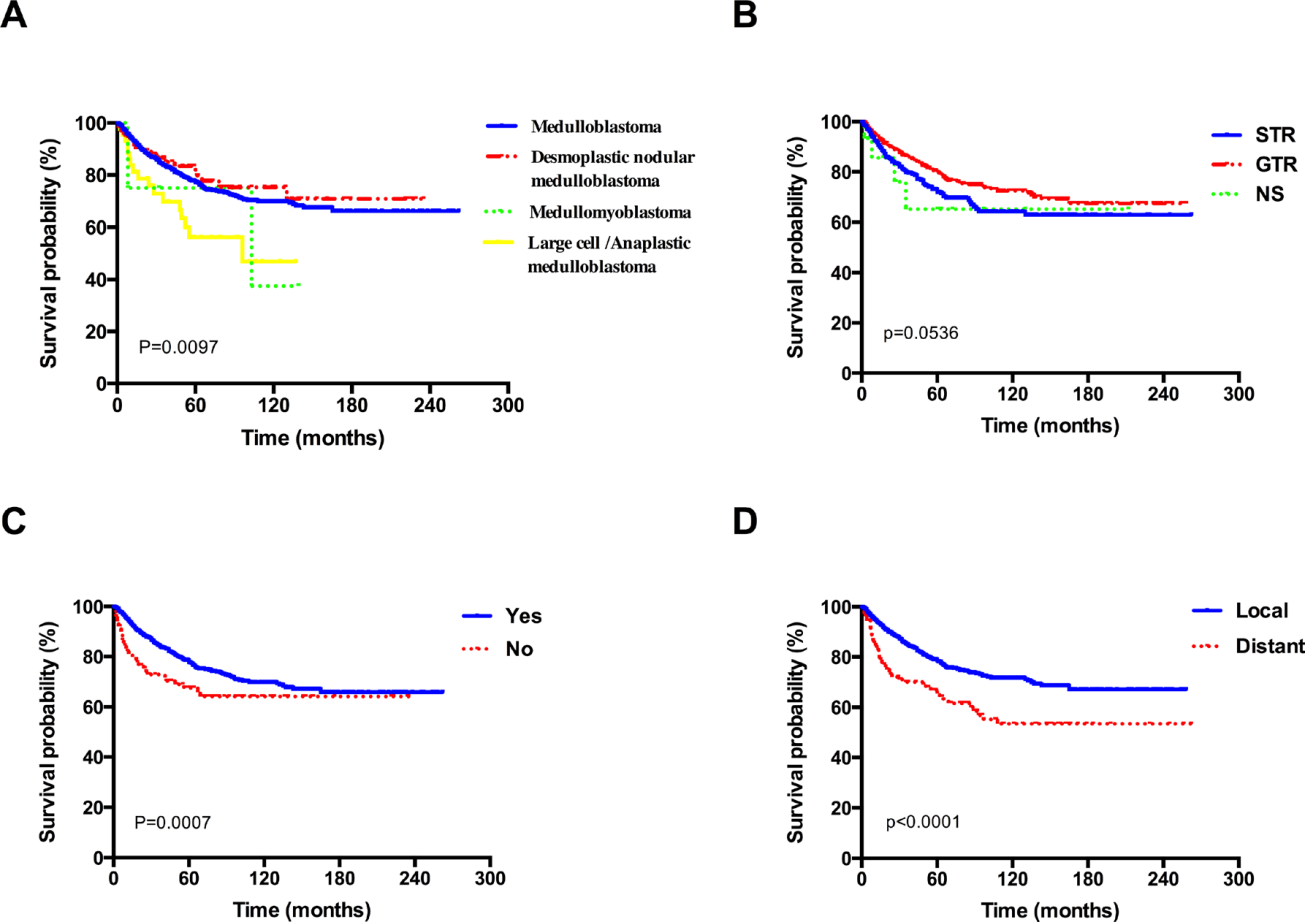
Kaplan–Meier overall survival curves stratified by (**A**) histological type, (**B**) extent of resection, (**C**) radiotherapy, (**D**) extent of disease at diagnoses for patients with MB.

As shown in Table 2, for all data gathered on patients, univariate survival analysis of clinical variables were evaluated with log-rank test. Histological type (Figure 3A), extent of resection (Figure 3B), radiotherapy (Figure 3C), extent of disease at diagnoses (Figure 3D) and year of diagnosis affected survival significantly (*P* < 0.05), while age at diagnosis, sex, race, marital status, size, and primary site showed no significant association with survival (*P* > 0.05).

**Table 2:** Univariate analysis on the impact of patient, tumor and management factors on overall survival

	Overall survival			
	HR	95% CI	Log-rank *P* value	Comparison group
**Age**				
**4-19**	0.90	0.69–1.16	0.411	**>19**
**Sex**				
**Female**	1.17	0.90–1.51	0.240	**Male**
**Race**				
**White**	1.09	0.75–1.59	0.659	**Non–white**
**Marital status**				
**Married**	0.86	0.61–1.21	0.381	**Unmarried**
**Histology**				
**Desmoplastic nodular MB**	0.89	0.56–1.41	0.611	**MB**
**Medullomyoblastoma**	1.95	0.49–7.87	0.347	
**Large cell /Anaplastic MB**	2.12	1.32–3.39	0.002	
**Extent of resection**				
**GTR**	0.71	0.54–0.92	0.009	**STR**
**NS**	1.82	0.85–3.92	0.125	
**Radiotherapy**				
**RT**	0.41	0.30–0.56	<0.001	**No RT**
**Year of diagnosis**	0.95	0.93–0.98		
**2003-2013**	0.68	0.52–0.88	0.004	**1992–2002**
**Size (mm)**	1.00	0.98–1.01	0.523	
**>40**	0.76	0.57–1.02	0.067	**≤40**
**Site**				
**Infratentorial**	0.73	0.43–1.26	0.255	**Supratentorial**
**Others**	1.80	0.87–3.73	0.114	
**Extent of disease at diagnoses**				
**Distal metastases (CSF, spinal cord or extraneural)**	1.79	1.30–2.48	<0.001	**Diseases confined to the brain**

Abbreviations: GTR: gross total resection; STR: subtotal resection; NS: no surgery; RT: radiotherapy; HR: hazard ratio; CI: confidence interval.

### Multivariate analysis

Multivariate analysis performed with the Cox proportional hazards regression model indicated that when analyzing for all patients, radiotherapy, histology, and extent of disease at diagnosis were the independent prognostic factors of survival (*P* < 0.05). Radiation utilization was significantly associated with reduced hazard of mortality (HR 0.41, 95% CI 0.28–0.60); whereas LC/A subtype (HR 2.31, 95% CI 1.34–3.97) and distal metastases (CSF, spinal cord or extraneural) (HR 1.66, 95% CI 1.12–2.48) were of poor prognosis. When the analysis was restricted to children, we found it interesting that similar results were observed except for the LC/A subtype (HR 1.95, 95% CI 1.00–3.83). However, in the adult group, different results were noted when comparing with children. LC/A subtype consistently associated with poor survival (HR 4.33, 95% CI 1.69–11.11). GTR was associated with good prognosis (HR 0.56, 95% CI 0.35 –0.90), while CSF, spinal cord, or extraneural metastases failed to show any statistical significance (HR 1.33, 95% CI 0.59–3.01). Radiotherapy was the only identical prognostic factor observed between children (HR 0.36, 95% CI 0.22–0.60) and adults (HR 0.47, 95% CI 0.26–0.84), which was associated with better survival.

## DISCUSSION

This study has compared the survival, clinical characteristics, and prognostic factors of children and adults with MB in a large series of patients contemporarily, showing that children and adults may have very similar outcomes, while the prognostic factors in adult MB are not comparable to those in children. Radiotherapy was the only identical prognostic factor observed in the two groups. In addition, distal metastases was a significant negative prognostic factor restricted to children, while GTR was a favorable prognostic factor, and LC/A histologic subtype was associated with poor survival for adults.

The 5- year overall survival rate for children is 75.5%. This is higher than the reported 50% by Karoly *et al.* in a series of 80 cases [12], but it is comparable to those who reported approximately 70% in other series of children [13]. The estimated 5- and 10-year survival rates for adults are 74.2% and 67.3% respectively, which are higher than the reported 72% and 55% in the French multi-center cohort of 253 patients [14]. Nevertheless, no significant difference of survival was observed between the two groups in our cohort, which was consistent with previously reported data, indicating that the survival was not affected by age at tumour onset [15] (Figure 1). However, our data did not support the extrapolation of Rose Lai’ s study that the relative survival of children with MB, especially in those older than 3 years of age, may be better than that in adults [6]. MB can affect individuals of all ages, which is common in children but is rare in adults [16]. Furthermore, our findings suggested that in the paediatric age group, MB occurred commonly in the first decade and in adults, the third decade, which was in accordance with the previous studies [3, 17–19]. In addition, the distribution of cases according to age at diagnosis showed an exponential decreasing trend (Figure 2). Notably, the CSF, spinal cord, or extraneural metastases, a worse clinical feature, was observed to be more frequent in children patients rather than in adults (Table 1), which may suggest a unique clinicobiological characteristic underlying children, in whom this tumor commonly occurs. In addition, there were more pediatric MB after 2003 (Table 1), which may be largely due to diagnostic improvements in brain-tumor imaging [3].

Our study confirmed that LC/A subtype and distal CSF, spinal cord, or extraneural metastases were the main adverse prognostic factors when analyzing all MB patients, while radiotherapy was correlated with a better prognosis (Table 3). When the analysis was restricted to children, LC/A subtype no longer had prognostic value, which was inconsistent with the previous studies that suggested children LC/A subtype was associated with an impaired prognosis of patients and inferior survival rates [20–22]. Given the low incidence of LC/A medulloblastoma, numbers of patients with this MB variant described in the literature were only limited. In addition, some investigators also suggested that patients with LC/A subtype can have favorable survival rates if clinical risk factors as metastases and young age are absent [23]. Thus this discrepancy might mainly because that the population in our study is relatively different compared to the previous studies. Likewise, when the analysis was restricted to adult, the LC/A subtype was shown to carry the worst prognosis among all histologic subtypes, while distal metastases was of no statistical significance in the multivariate model. The discrepancy in outcomes with distant metastases which might be associated with the different tumourigenic mechanisms involved in the development of MB [24], patients with MB in adults and children are histologically and genetically different diseases, with more mutations being observed in children [25, 26]. Furthermore, adult tumors were biologically less aggressive, having lower growth rate parameters as compared to childhood tumors [27]. These results again agreed with previous research [6]. Another discrepancy with children is that GTR was associated with a better outcome in adult group (Table 3). Which may be attributable to that childhood tumors were more commonly of midline cerebellar vermis, [28, 29], many MB have an attachment to the floor of the fourth ventricle or brainstem, aggressive initial surgical resection can greatly increase surgical complications [30–32]. Thompson *et al.* have also identified that there is no overall survival benefit for GTR over STR [32]. While the lateral location occurred more frequently in adults [28], which enabled total resection of tumors without affecting the function of critical structures, resulting in improved long-term survival [11, 33]. In addition, adult MB patients are more amenable to complete surgical clearance than children. Rose Lai and Chan’s group also reported similar results [6, 8]. However, the complete resection in 1 study only resulted in severely reduced postoperative performance status [7].

**Table 3:** Multivariate analysis of patient and treatment factors in MB patients diagnosed from 1992 to 2013

Variables	All patients		Children		Adult		Comparison group
	HR (95% CI)	*P*	HR (95% CI)	*P*	HR (95% CI)	*P*	
Histology							
Desmoplastic nodular MB	0.87 (0.49–1.54)	0.628	0.31 (0.10–1.00)	0.050	1.82 (0.91–3.63)	0.089	MB
Medullomyoblastoma	2.54 (0.61–10.48)	0.198	2.81 (0.66–11.98)	0.163	0.00 (0.00–4.12E+194)	0.972	
Large cell /Anaplastic MB	2.31 (1.34–3.97)	0.003	1.95 (1.00–3.83)	0.051	4.33 (1.69–11.11)	0.002	
Extent of resection							
GTR	0.76 (0.56–1.03)	0.078	0.96 (0.64–1.43)	0.822	0.56 (0.35–0.90)	0.016	STR
NS	0.37 (0.05–2.68)	0.325	0.00 (0.00–2.18E+195)	0.962	0.71 (0.10–5.34)	0.742	
Radiotherapy							
RT	0.41 (0.28–0.60)	<0.001	0.36 (0.22–0.60)	<0.001	0.47 (0.26–0.84)	0.011	No RT
Size (mm)							
>40	0.80 (0.59–1.09)	0.150	0.77 (0.52–1.15)	0.200	0.88 (0.54–1.43)	0.611	≤40
Extent of disease at diagnoses							
Distal metastases (CSF, spinal cord or extraneural)	1.66 (1.12–2.48)	0.012	1.86 (1.17–2.97)	0.009	1.33 (0.59–3.01)	0.490	Diseases confined to the brain
Year of diagnosis 2003–2013	0.74 (0.53–1.03)	0.072	0.75 (0.48–1.17)	0.206	0.68 (0.41–1.12)	0.128	1992–2002

Abbreviations: GTR: gross total resection; STR: subtotal resection; NS: no surgery; RT: radiotherapy; HR: hazard ratio; CI: confidence interval.

In a previous multicenter, retrospective study on MB, certain prognostic factors in adults have been demonstrated to be similar to those observed in children such as brainstem, floor of fourth ventricle involvement, and dose to the posterior cerebral fossa [14]. Notably, in our study, craniospinal irradiation, a main treatment modality for MB, was demonstrated to be the only identical prognosis factor of the two groups, which was correlated with a longer survival. Nevertheless, these results cannot be considered conclusive, since the efficacy and patients’ survival were determined and influenced by the quality of the regimens and the dose administered to different targets. For example, radiation dose to the posterior fossa less than 50 Gy has been reported to be a poor prognostic factor [14], while a high radiation dose to the spinal cord was correlated with a better outcome [7]. In addition, cumulated doses were heterogeneous. Unfortunately, the SEER data did not provide information on details of radiation dosage.

Despite the fact that the survival of this disease would be improved due to enhanced imaging technologies, improved surgical planning, and radiation techniques, it is worth noting in this cohort year of diagnosis was not found to be a prognostic factor. In addition, in this study, tumor size was unable to be established for prognostic value, which again appeared to be consistent with the findings of another series [6].

Some major limitations in the current study due to intrinsic limitations in the SEER database made the interpretation of the results difficult. First, no central review of the histologic specimens could be performed. Second, inadequate data, such as total dose, fraction size, radiation ports, and radiation volume were provided in the SEER registry. Third, even though the four core subtypes of MB, including WNT, SHH, Group 3, and Group 4, have been demonstrated to be correlated with patients’ survival and response to treatment [34], information on genetic profiles were unavailable. Forth, information regarding various clinical variables, such as patient performance status and duration of symptoms were unavailable. Last, lack of information on chemotherapy regimens, which might potentially confound our results.

In conclusion, this preliminary study based on SEER database helped to determine some basic survival parameters and prognostic factors. Despite the fact that children patients tend to exist more distal metastases, and did not show any survival benefit when treated with GTR as opposed to the adults, children and adult patients with MB did not differ with respect to the overall survival. Radiotherapy was demonstrated to be the only identical prognostic factor, that could reduce the fatal risk of MB, for both children and adult patients.

## MATERIALS AND METHODS

### Patient population

We performed a retrospective analysis using SEER Program (www.seer.cancer.gov) and SEER*Stat Database: Incidence-SEER 18 Registries Limited Use, November 2015 Submission (1973–2013), National Cancer Institute, Division of Cancer Control and Population Sciences (DCCPS), Surveillance Research Program, Cancer Statistics Branch, released April 2016, based on the November 2015 submission [35]. The SEER database provides information about the patients’ demographic characteristics, type of malignancy, treatment protocols, and clinical information at the time of diagnosis. Patients were followed-up annually for survival status. We extracted the data of patients with MB cases from SEER 18 registries using the following parameters: age > 3 years old, International Classification of Disease in Oncology 3rd edition (ICD-O3) code 9470, 9471, 9472, and 9474, primary site in the cerebellum and other clinically relevant. Patients without histological confirmation, patients with death certificate only, and patients with multiple primary tumors without active follow-up or explicit survival results were excluded from the survival study to improve the accuracy of survival analyses. The study period was 1992–2013. Information was obtained about their demographic characteristics, year of diagnosis, and duration of survival. However, the data on patient symptoms, performance status, and treatments such as adjuvant chemotherapy are not available in the database. Also this research did not involve interaction with human subjects or the use of any personal identifying information. Hence, it was exempted from the institutional review board approval. Informed consent was not applicable to this study.

### Patient population and variable collection

There were 965 patients studied who were diagnosed with MB between 1992 and 2013. Among the demographic variables, according to the age at diagnosis, data were compared with children (between 4 and 19 years of age) and adults (≥20 years). Demographic and clinical variables included sex (male, female), race/ethnicity (white, non-white), marital status at diagnosis (married, unmarried, unknown), histologic subtype (medulloblastoma, desmoplastic nodular medulloblastoma, medullomyoblastoma, and large cell /anaplastic (LC/A) medulloblastoma), tumor size (by category: ≤4 cm, >4 cm), year of diagnosis (1992–2002, 2003–2013), extent of surgery (gross total resection, subtotal resection, or no surgery), and radiotherapy status (radiotherapy, no radiotherapy), primary tumor sites (supratentorial, infratentorial, others), extent of disease at diagnoses (diseases confined to the brain, distal metastases to CSF, spinal cord or extraneural), when applicable, were also recorded. Data regarding the use of adjuvant chemotherapy was not available in the SEER database.

### Statistical analysis

Statistical analysis was performed using SPSS statistical software version 19.0 (SPSS Inc., IBM Corporation, Chicago, IL, USA). Significant differences in patient demographics and tumor characteristics between the two groups were detected using the Pearson Chi-square test and the Wilcoxon rank sum test. Overall mortality was chosen as an endpoint. Survival was calculated using the Kaplan-Meier method and the log-rank test was used to compare survival curves. Univariate and multivariate models were established to evaluate correlations between various covariates and survival. Those variables that achieved a *P*-values < 0.1 were included in a multivariable Cox proportional hazard model. We reported our findings using hazard ratios (HRs), 95% confidence intervals (CIs), and *P* values. Two-sided *P*-values < 0.05 were considered statistically significant.

## Abbreviations

CNS: central nervous system
CI: confidence intervals
CSF: cerebrospinal fluid
GTR: gross total resection
HR: hazard ratio
HGG: high-grade glioma
MB: medulloblastoma
NCI: National Cancer Institute
NS: no surgery
RT: Radiotherapy
STR: subtotal resection
SEER: Surveillance, Epidemiology, and End Results
